# Genome-wide identification of growth−regulating factor and *GRF*−interacting factor gene families across three Solanaceae species with functional analysis in pepper regeneration

**DOI:** 10.3389/fpls.2025.1684045

**Published:** 2025-12-10

**Authors:** Yanping Wang, Li Jia, Han Wang, Xiujuan Zhou, Congsheng Yan, Qiangqiang Ding, Xiujing Hong, Yanxiang Dong, Yan Wang, Haikun Jiang

**Affiliations:** 1 Institute of Vegetables, Anhui Academy of Agricultural Sciences, Hefei, China; 2 Key Laboratory of Horticultural Crop Germplasm Innovation and Utilization (Co-Construction by Ministry and Province), Institute of Horticulture, Anhui Academy of Agricultural Sciences, Hefei, China; 3 Anhui Provincial Key Laboratory for Germplasm Resources Creation and High-Efficiency Cultivation of Horticultural Crops, Institute of Vegetables, Anhui Academy of Agricultural Sciences, Hefei, China

**Keywords:** *GRF*, *GIF*, gene families, pepper, regeneration

## Abstract

The growth-regulating factor (*GRF*) and *GRF*-interacting factor (*GIF*) gene families play crucial roles in enhancing plant genetic transformation efficiency. However, the functional contributions of pepper *GRF* and *GIF* genes in improving transformation efficiency remain unexplored. In this study, we identified 31 *GRF* genes and 9 *GIF* genes across pepper, tomato, and tobacco. A systematic analysis was conducted to characterize their phylogenetic relationships, gene structures, collinearity, and expression patterns. Collinearity analysis revealed large-scale duplication events among these three Solanaceae species. Furthermore, qRT-PCR analysis demonstrated significantly elevated ex-pression levels of pepper GRF and GIF genes in regenerative tissues, indicating their critical functional roles during pepper regeneration processes. Our findings provide novel insights into the evolution of *GRF* and *GIF* gene families in Solanaceous plants and identify several key candidate genes for future functional studies aimed at enhancing pepper regeneration efficiency and genetic transformation.

## 1 Introduction

The growth-regulation factor (*GRF*) and *GRF*-interaction factor (*GIF*) genes were a family of plant-specific transcription factors, where GRF proteins binded to DNA and regulated the expression of other genes, and GIF interacted with GRF to form a functional transcription complex to regulate its activity (Horiguchi et al., 2005). GRF proteins were characterized by two conserved N-terminal domains: the QLQ domain and the WRC domain (Kim et al., 2003; Choi et al., 2004). The WRC domain binded to cis-regulatory elements of target genes to modulate their expression, while the QLQ domain mediated interaction with GIF proteins to form functional transcriptional activator complexes (Wang et al., 2023a). GIF proteins feature conserved SSXT structural domains (Kim and Kende, 2004) and exhibited robust transcriptional activation potential along with cell proliferation promoting activity. Together with their interacting GRF partners, these proteins played pivotal roles in regulating plant growth and developmental processes (Lee et al., 2009, Lee et al., 2014).

The *GRF-GIF* complex played a critical regulatory role in cell reprogramming during plant regeneration (Zhang et al., 2020). The *TaGRF4-GIF1* complex significantly enhanced transformation efficiency in wheat, reducing the regeneration timeline from 91 to 56 days (Debernardi et al., 2020). In sorghum, both *GRF4-GIF1* co-expression and *GRF5* overexpression significantly improved genetic transformation efficiency, reducing the total process duration to under two months (<60 days) (Li et al., 2024). In maize, co-expression of *ZmGRF1-ZmGIF1* enhanced transformation efficiency 3.5-6.5fold, while maintaining normal plant growth phenotypes (Vandeputte et al., 2024). The above results demonstrated the applicability of the *GRF-GIF* complex across diverse monocot species without significant pleiotropic effects. In dicot plants, the *GRF-GIF* complex also exhibited an analogous function. In soybean, expression of the *GRF3-GIF1* chimera significantly improved re-generation and transformation efficiency and increased the number of transformable varieties; moreover, *GmGRF3-GIF1* could be combined with CRISPR/Cas9 for efficient gene editing (Zhao et al., 2025). Other *GRF-GIF* fusion proteins have also been successfully utilized to enhance transformation efficiency. For instance, *Arabidopsis AtGRF5* has been shown to improve transformation efficiency in sugar beet, soybean, sunflower, and maize. Similarly, *GRF4-GIF1* from tomato, grape, and citrus have all demonstrated the ability to enhance transformation efficiency in lettuce (Bull et al., 2023). Based on the above results, it could be concluded that the *GRF-GIF* complex exhibited broad applicability across different genotypes and demonstrated no significant functional divergence among diverse species.

Currently, functional characterization of genes in pepper (Capsicum annuum) primarily relies on virus-induced gene silencing (VIGS) and heterologous overexpression in other species. However, a limited number of studies have reported the establishment of stable genetic transformation systems in pepper. The Tomato spotted wilt virus (TSWV)-based system enables efficient CRISPR/Cas9-mediated genome editing in pepper, with up to 77.9% of regenerated plants shown to carry heritable mutations (Zhao et al., 2024). Optimization of the transformation protocol by using cotyledon explants with vacuum infiltration and omitting the pre-culture step significantly enhanced transformation efficiency, achieving an effective rate of approximately 5% (Tang et al., 2025). Supplementation of the culture medium with the small peptide CaREF1 further improved transformation, resulting in 100% gene editing efficiency in the T0 generation (Wang et al., 2025).

Notably, the application of the GRF–GIF complex in the development of a genetic transformation system for pepper has not yet been reported. This study aims to lay the foundation for constructing a highly efficient pepper transformation system by analyzing the evolutionary relationships of GRF and GIF genes in Solanaceae and investigating their expression profiles during pepper regeneration.

## 2 Results

### 2.1 Identification of *GRF* and *GIF* gene families in pepper, tomato and tobacco

This study identified 7 *GRF* genes in pepper (**Table 1**) and 12 *GRF* genes each in tomato and tobacco (**Supplementary Tables S1**, **S2**). Comprehensive gene characteristics including chromosomal locations, exon-intron structures, genomic lengths, predicted isoelectric points (pI), molecular weights, and subcellular localization were presented in the respective tables. The pepper GRF genes were present on four of the 12 chromosomes (Chr1, Chr2, Chr3, Chr4), with the number of exons ranging from 3-5 (*CaGRF4- CaGRF7*), amino acid number ranging from 311aa-596aa (CaGRF4- CaGRF1), isoelectric point size ranging from 5.94-9.11 (CaGRF5-CaGRF4), molecular weight size between 34KDa-64KDa (CaGRF4-CaGRF1). The tomato GRF genes were present on 7 of the 12 chromosomes (Chr2, Chr3, Chr4, Chr7, Chr8, Chr9, Chr10), with the number of exons ranging from 1-6 (*SlGRF9-SlGRF7*), amino acid number ranging from 468aa-1788aa (SlGRF9- SlGRF1), isoelectric point sizes between 5.97-9.53 (SlGRF10- SlGRF3), molecular weight size be-tween 17KDa-64KDa (SlGRF9- SlGRF1). Tobacco GRF genes were present on 4 of the 12 chromosomes (Chr1, Chr4, Chr5, Chr10), with the number of exons ranging between 3-5 (*NtGRF8-NtGRF11*) amino acid number ranging between 324aa-605aa (NtGRF9- NtGRF1), isoelectric point size ranging between 5.57-9.17 (NtGRF10- NtGRF12), molecular weight size between 36KDa-65KDa (NtGRF9- NtGRF2). The subcellular localization of the above genes was predicted to be localized in the nucleus.

**Table 1:** Information of *GRFs* in pepper.

Gene name	GeneID	Chr.	Exon number	CDS (bp)	Protein length (aa)	Theoretical pI	Mw (KDa)	Subcellular localization
*CaGRF1*	*Capana04g000709*	4	4	1791	596	7.99	64	Nucleus
*CaGRF2*	*Capana02g002938*	2	4	1713	570	8.42	61	Nucleus
*CaGRF3*	*Capana01g000919*	1	4	1203	400	8.94	44	Nucleus
*CaGRF4*	*Capana01g004288*	1	3	936	311	9.11	34	Nucleus
*CaGRF5*	*Capana03g001909*	3	4	1473	490	5.94	53	Nucleus
*CaGRF6*	*Capana01g000039*	1	4	1317	438	6.39	47	Nucleus
*CaGRF7*	*Capana01g001420*	1	5	1209	402	7.94	44	Nucleus

The *GIF* gene family exhibited a relatively limited number of members. There were 3 *GIF* genes in pepper, and 4 and 2 *GIF* genes in tomato and tobacco, respectively. Detailed information on chromosomal localization, number of exons, gene length, and isoelectric point subcellularity were shown in **Table 2** and **Supplementary Tables S3, S4**.

**Table 2:** Information of *GIFs* in pepper.

Gene name	GeneID	Chr.	Exon number	CDS (bp)	Protein length (aa)	Theoretical pI	Mw (KDa)	Subcellular localization
*CaGIF1*	*Capana12g002119*	12	6	735	244	7.13	26	Nucleus
*CaGIF2*	*Capana05g000500*	5	4	648	215	6.17	23	Nucleus
*CaGIF3*	*Capana04g001425*	4	1	504	167	4.74	18	Nucleus

### 2.2 Phylogenetic and structural analysis of the *GRF* and *GIF* gene families of pepper, tomato and tobacco

**Figure 1:**
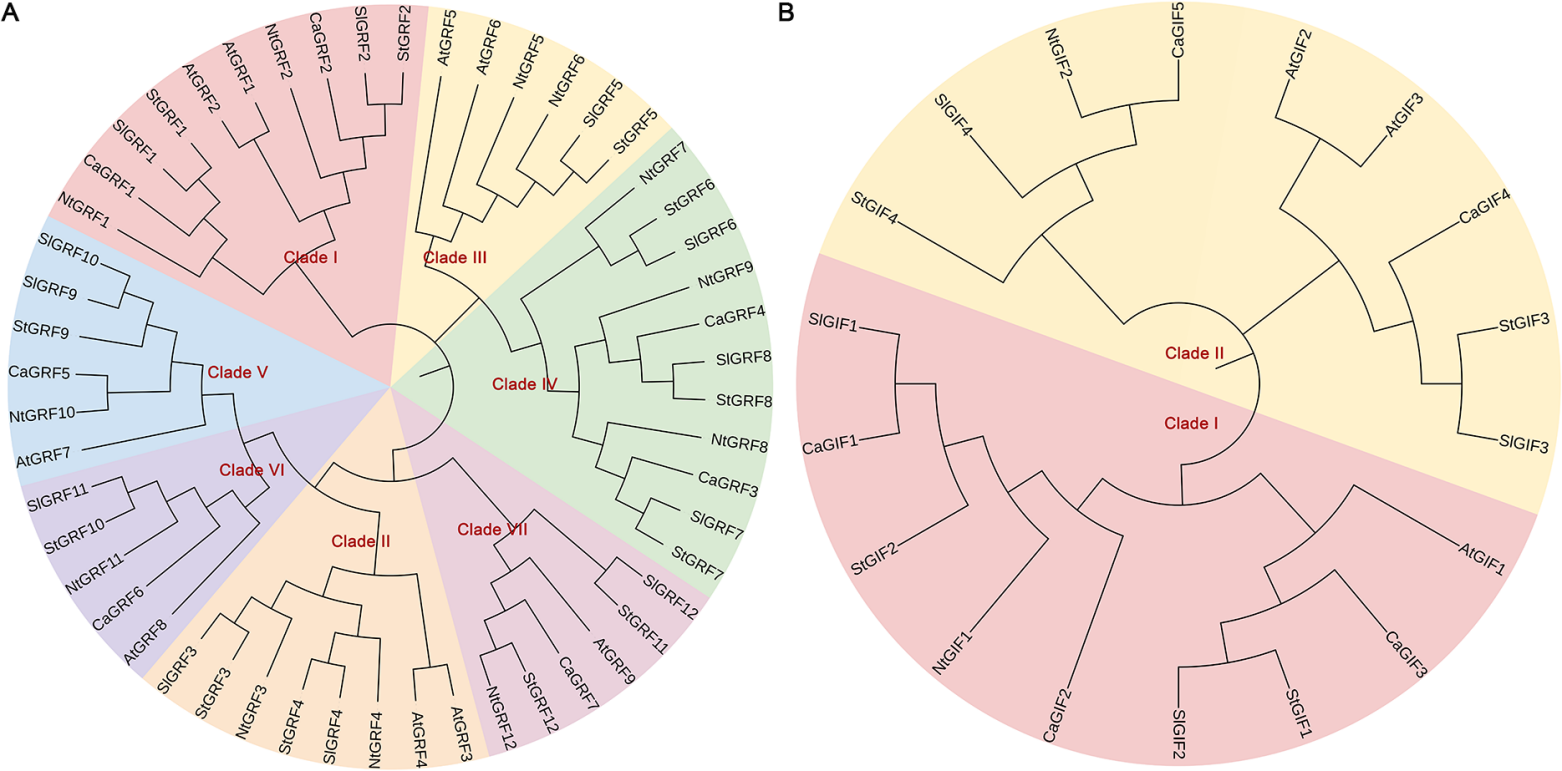
Phylogenetic analysis of GRF and GIF proteins. **(A)** Phylogenetic analysis of GRFs. **(B)** Phylogenetic analysis of GIFs. The protein sequences of GRF and GIF from Arabidopsis, pepper, tomato, and tobacco were aligned using the CLUSTALW multiple sequence alignment tool. At, *Arabidopsis thaliana*; Ca, *Capsicum annuum*; Sl, *Solanum lycopersicum*; St, Solanum tuberosum; Nt, *Nicotiana tabacum*.

Phylogenetic analysis was performed to examine the evolutionary relationships of *GRF* and *GIF* gene families among *Arabidopsis thaliana*, *Capsicum annuum*, *Solanum lycopersicum*, *Solanum tuberosum* and *Nicotiana tabacum* (**Figure 1**). Based on the phylogenetic clustering of Arabidopsis thaliana, the *GRF* gene family was classified into seven distinct subfamilies, comprising between two and eight members per subfamily. Notably, no pepper genes were identified within Clade II or Clade III (**Figure 1A**). It is speculated that the pepper genes in these clades may have been lost during evolution under environmental influences. Phylogenetic analysis divided the GIF gene family into two distinct subfamilies, with each subfamily containing nine members (**Figure 1B**).

**Figure 2:**
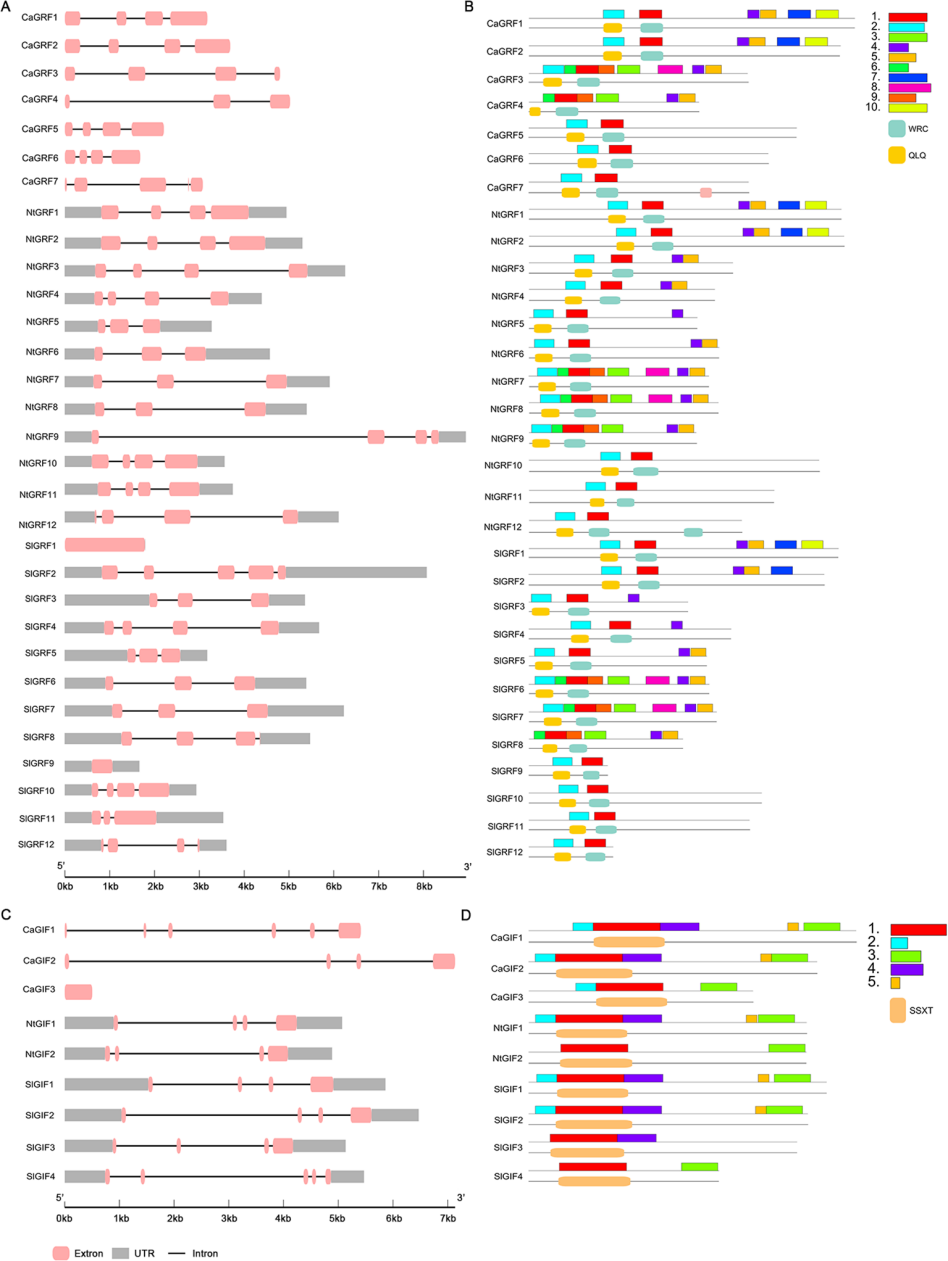
Gene structure motif and conserved domains of the GRF and GIF gene families. **(A)** Gene structure of *GRF* gene family. **(B)** Motif and conserved domains analysis of GRF gene family. **(C)** Gene structure of *GIF* gene family. **(D)** Motif and conserved domains analysis of GIF gene family.

A comprehensive analysis of the gene structures, protein motifs, and conserved domains of the GRF and GIF families revealed that all genes from the three Solanaceae species contain UTR regions, with the exception of the pepper GRF genes and tomato SlGRF1 (**Figures 2A, C**). Motif composition analysis showed significant differences among GRF clades (**Figures 2B, D**). Except for CaGRF4 and SlGRF8, all proteins contained motif 1 and motif 2. Additionally, clade I included motifs 4, 5, 7, and 10; clades II and III both contained motif 4; clade IV was composed of motifs 3, 4, 5, 6, and 9; while clades V–VII possessed only motifs 1 and 2 (**Figure 2B**). Analysis of conserved protein domains indicated that the QLQ and WRC domains correspond to motif 1 and motif 2, respectively. Phylogenetic analysis classified the GIF gene family into two subfamilies: clade I, which contains all five conserved motifs (1–5), and clade II, which retains only motifs 1 and 3 (**Figure 2D**). Conserved domain analysis showed that the SSXT domain corresponds to motif 1. Detailed motif sequences are provided in **Supplementary Tables S5, S6**.

### 2.3 Duplication events of *GRF* and *GIF* genes among pepper, tomato, and tobacco

**Figure 3:**
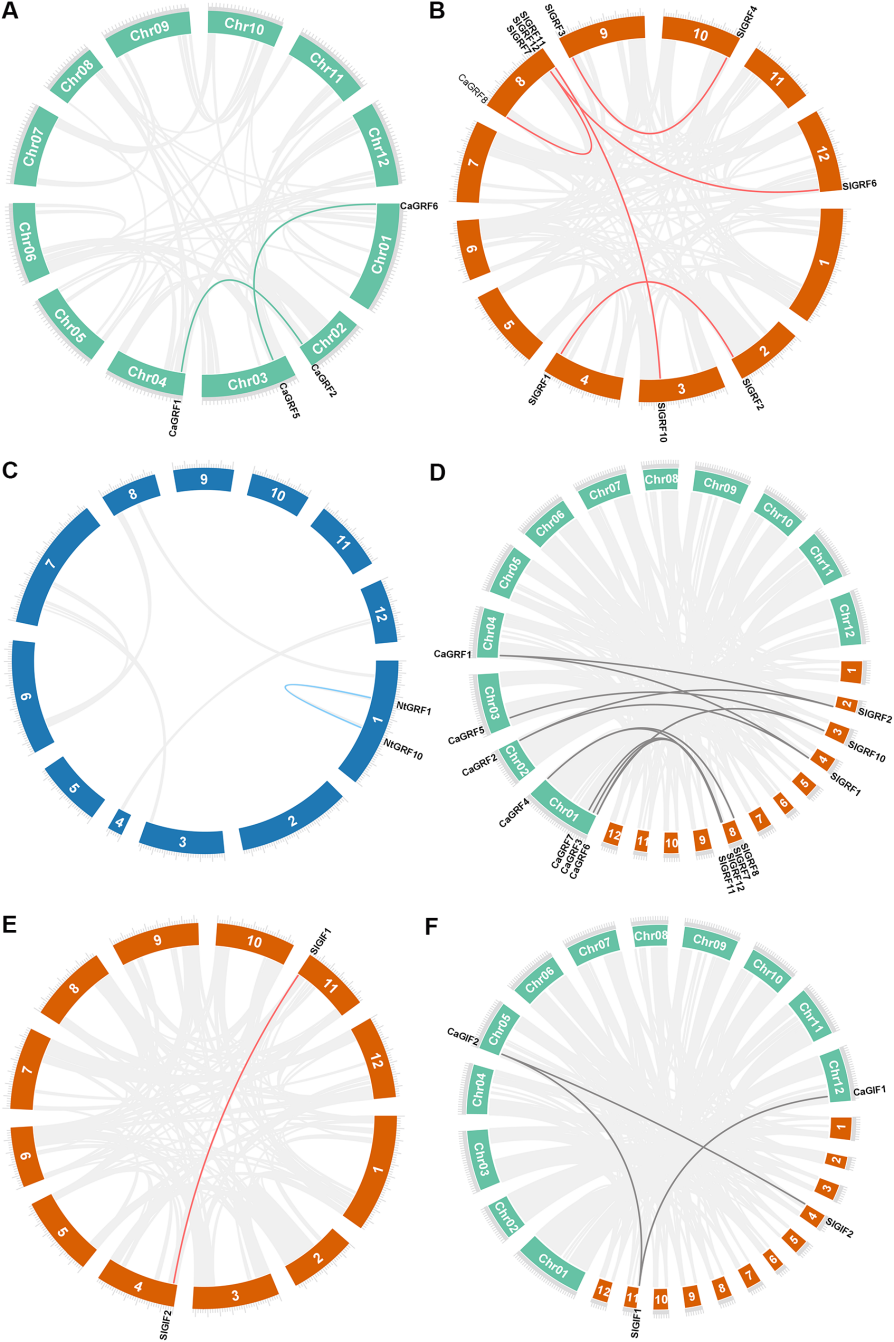
Synteny of three plants GRF and GIF genes. **(A)** Duplication events of GRF genes in pepper. **(B)** Duplication events of GRF genes in tomato. **(C)** Duplication events of GRF genes in tobacco. **(D)** Duplication events of GRF genes in pepper and tomato. **(E)** Duplication events of GIF genes in tomato. **(F)** Duplication events of GIF genes in pepper and tomato. Lines indicated duplicated GRFs and GIFs gene pairs. Green: pepper chromosomes. Orange: tomato chromosomes. Blue: tobacco chromosomes.

Genome-wide analysis of the three Solanaceous species revealed duplicated GRF gene pairs in each species. Pepper contained two duplicate pairs (*CaGRF1/CaGRF2* and *CaGRF5/CaGRF6*) (**Figure 3A**), while tomato showed five duplicated pairs (SlGRF1/SlGRF2, SlGRF3/SlGRF4, *SlGRF6/SlGRF7*, *SlGRF7/SlGRF8*, and *SlGRF10/SlGRF11*) (**Figure 3B**). Tobacco contained one duplicated pair (*NtGRF10/NtGRF12*) (**Figure 3C**). To clarify GRF genes evolutionary relationships across species, we performed comparative analyses. Due to tobacco’s tetraploid complexity, covariance analysis was restricted to tomato and pepper. Comparative analysis identified 11 orthologous GRF gene pairs between pepper and tomato: *CaGRF1/SlGRF1*, *CaGRF1/SlGRF2*, *CaGRF2/SlGRF1*, *CaGRF2/SlGRF2*, *CaGRF3/SlGRF7*, *CaGRF4/SlGRF7*, *CaGRF4/SlGRF8*, *CaGRF5/SlGRF10*, *CaGRF6/SlGRF10*, *CaGRF6/SlGRF11*, and *CaGRF7/SlGRF7* (**Figure 3D**). Notably, four tomato GRF genes (*SlGRF3*, *SlGRF4*, *SlGRF5*, *SlGRF6*) were absent from all duplication events. The presence of collinearity suggested that homologous gene pairs in pepper and tomato may retain similar functions. Previous studies had reported that all GRF genes in tomato are highly expressed in meristematic tissues. In particular, SlGRF6 (also referred to as SlGRF4 or SlGRF4c in some studies) had been demonstrated to significantly enhance the genetic transformation efficiency in tomato (Swinnen et al., 2025; Khatun et al., 2017). This key finding in tomato provided an important theoretical foundation for functional studies and genetic improvement applications of its homologous genes in pepper.

In the GIF gene family, neither pepper nor tobacco contained duplicated gene pairs, while tomato retained only one duplicated pair (*SlGIF1/SlGIF2*) (**Figure 3E**). Comparative synteny analysis between pepper and tomato identified three conserved orthologous gene pairs (*CaGIF1/SlGIF1*, *CaGIF2/SlGIF1* and *CaGIF2/SlGIF2*) (**Figure 3F**).

The ratio of non-synonymous to synonymous substitutions (Ka/Ks) was used to calculate evolutionary selection. Purifying selection was inferred when the ratio Ka/Ks of a pair of sequences was less than one (Wang et al., 2023b). In all three Solanaceae, selection analysis showed that the replicated gene pairs were subjected to purifying selection (**Table 3**).

**Table 3:** Evolutionary selection between *GRFs* and *GIFs* in three plants.

Seq 1	Seq 2	Ka	Ks	Ka/Ks
*CaGRF1*	*CaGRF2*	0.422484583	0.113246857	0.181623149
*CaGRF1*	*SlGRF1*	0.047895799	0.278757811	0.171818682
*CaGRF1*	*SlGRF2*	0.466670051	2.013507064	0.231769761
*CaGRF2*	*SlGRF1*	0.426705586	2.298026438	0.185683497
*CaGRF2*	*SlGRF2*	0.096340461	0.232375096	0.414590298
*CaGRF3*	*SlGRF7*	0.025867287	0.210058104	0.123143485
*CaGRF4*	*SlGRF7*	0.110940521	0.654306443	0.169554377
*CaGRF4*	*SlGRF8*	0.051246481	0.179036877	0.286234216
*CaGRF5*	*CaGRF6*	0.623770458	2.328745694	0.267856838
*CaGRF5*	*SlGRF10*	0.139987344	0.318356601	0.439718681
*CaGRF6*	*SlGRF10*	0.619660504	3.145532941	0.196996984
*CaGRF6*	*SlGRF11*	0.113246857	0.389496521	0.290751911
*CaGRF7*	*SlGRF7*	0.463757122	1.796551129	0.258137447
*SlGRF1*	*SlGRF2*	0.394685523	2.195419547	0.179776811
*SlGRF3*	*SlGRF4*	0.220894591	0.922546525	0.239440055
*SlGRF6*	*SlGRF7*	0.1279032739	0.589869118	0.216833311
*SlGRF7*	*SlGRF8*	0.1339763171	0.722929528	0.185324173
*SlGRF10*	*SlGRF11*	0.766514196	2.785525751	0.275177566
*NtGRF10*	*NtGRF12*	0.719498828	2.070650817	0.347474727
*CaGIF1*	*SlGIF1*	0.056168603	0.332930652	0.168709619
*CaGIF2*	*SlGIF1*	0.135680292	0.749248787	0.181088438
*CaGIF2*	*SlGIF2*	0.039846481	0.231410408	0.172189665
*SlGIF1*	*SlGIF2*	0.156542721	0.862878454	0.181419203

### 2.4 Cis-acting element analysis of the *GRFs* and *GIFs* promoters

**Figure 4:**
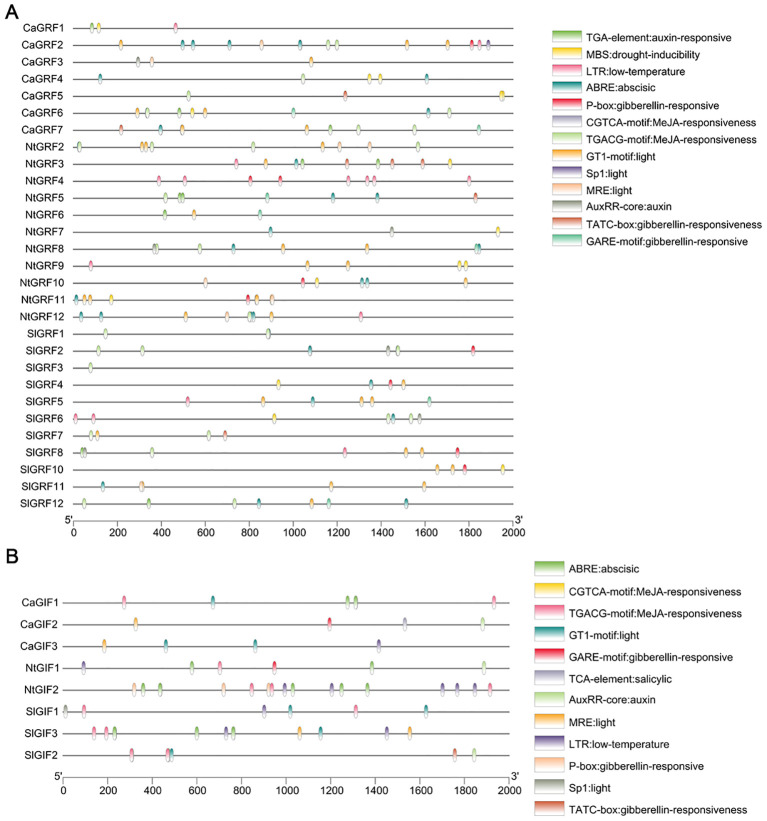
Analysis of promoter cis-acting elements. **(A)** Analysis of promoter cis-acting in three plant *GRF* genes. **(B)** Analysis of promoter cis-acting in three plant *GIF* genes.

To investigate potential roles in plant growth and development, we analyzed promoter cis-acting elements of *GRF* and *GIF* genes in the three Solanaceous species (**Figure 4**; **Supplementary Table S7**). The cis-acting elements of the 2000 bp upstream promoter were mapped through the RSAT website (http://rsat.eead.csic.es/plants/dna-pattern_form.cgi, accessed on 25 April 2025). Promoter analysis demonstrated that *GRF* and *GIF* genes in all three species harbor abundant cis-acting elements linked to hormonal responses (auxin, jasmonic acid, and gibberellin), environmental stresses (drought and low temperature), and light responsiveness, indicating their potential crucial roles in regulating plant adaptation to both biotic and abiotic stresses. Accumulating evidence demonstrates that GRF-GIF interactions substantially enhance plant transformation efficiency. Promoter analysis further reveals an enrichment of phytohormone-responsive cis-elements (particularly auxin and gibberellin), strongly supporting their functional importance in plant regeneration processes.

### 2.5 Expression patterns of *GRFs* and *GIFs* in regenerating tissues

**Figure 5:**
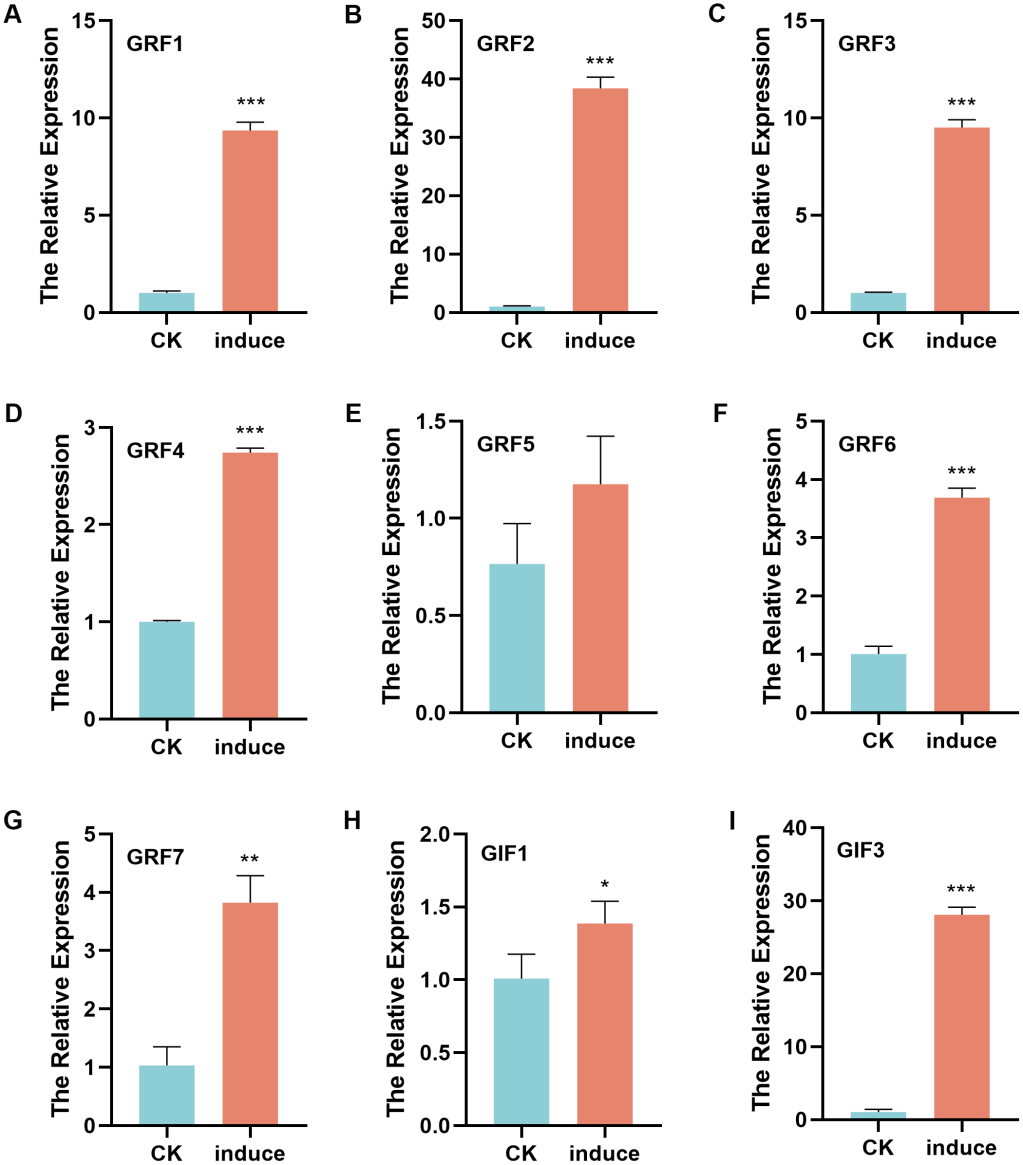
Relative expression of GRF and GIF genes in cotyledons and callus tissues of peppers. **(A)** The relative expression of GRF1. **(B)** The relative expression of GRF2. **(C)** The relative expression of GRF3. **(D)** The relative expression of GRF4. **(E)** The relative expression of GRF5. **(F)** The relative expression of GRF6. **(G)** The relative expression of GRF7. **(H)** The relative expression of GIF1. **(I)** The relative expression of GIF3. CK: cotyledons tissues. Induce: callus tissues (p*<0.05, p**<0.01, p***<0.001).

To examine *GRF* and *GIF* gene expression during regeneration, pepper Zunla-1 seeds were germinated until cotyledgers fully unfolded (**Supplementary Figure S2A**), with a subset of seed-lings directly sampled as controls while others were transferred to shoot regeneration culture medium. Callus tissues were subsequently collected after 14 days of culture for comparative analysis (**Supplementary Figure S2B**). Total RNA was isolated from both cotyledons and callus tissues for RT-qPCR analysis (**Figure 5**). All seven *GRF* genes showed significant upregulation in callus tissues compared to cotyledon controls, with *CaGRF2* exhibiting the most dramatic induction (39.3-fold). The relative expression levels of other *GRF* genes were: *CaGRF1* and *CaGRF3* (9.5-fold), *CaGRF7* (4-fold), *CaGRF6* (3.6-fold), and *CaGRF4* (2.7-fold). Three *GIF* genes showed differential expression during regeneration: *GIF2* was unexpressed, *GIF1* slightly upregulated (1.3-fold), while *GIF3* showed strong induction (28.4-fold). Combined with *GRF2*’s 39.3-fold upregulation, the *GRF2-GIF3* pair appears crucial for pepper regeneration, advancing Capsicum transformation research.

To investigate whether the non-significant expression of *CaGRF5* in regenerative tissues and the absence of CaGIF2 expression are related to tissue specificity, this study conducted a heatmap analysis of the expression profiles of the *GRF* and *GIF* gene families (**Supplementary Figure S3**). The results showed that, except for weak expression detected in seeds, *CaGRF5* expression was not observed in any other examined tissues. In contrast, *CaGIF2* exhibited a distinct high-expression pattern in fruits and seeds, indicating that *CaGIF2* may have significant tissue specificity.

### 2.6 Interaction analysis between GRF and GIF proteins

**Figure 6:**
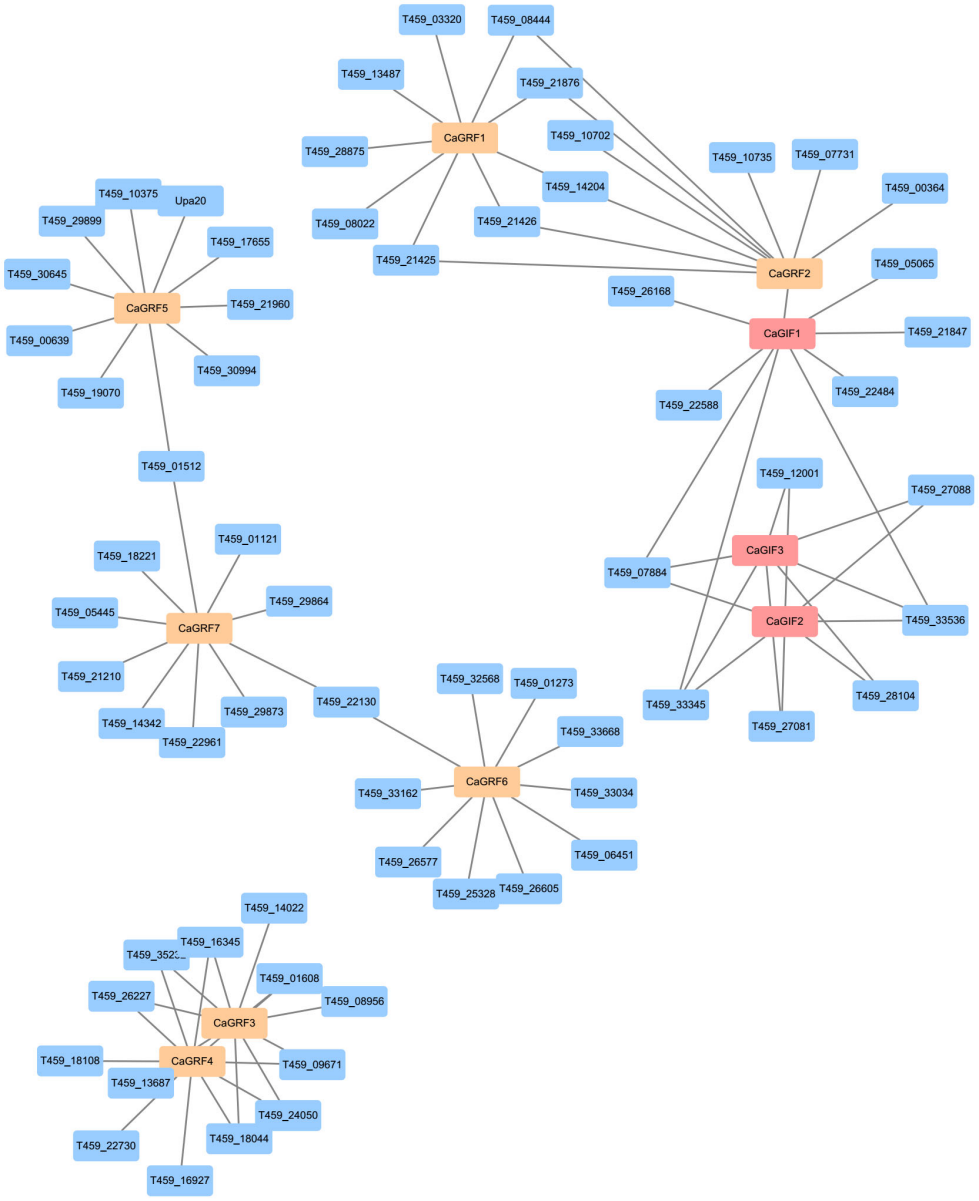
Predicted protein interaction network. The protein interaction network of CaGRF and CaGIF proteins was predicted based on the information with from the STRING database.

To investigate the interaction relationship between GRF and GIF proteins, the potential interaction between GRF and GIF proteins were predicted using the STRING database (Szklarczyk et al., 2019). The results indicated that all seven GRF proteins and three GIF proteins exhibited interactions with various other proteins. The prediction suggested potential interactions between CaGRF3 and CaGRF4, CaGIF2 and CaGIF3, as well as CaGRF2 and CaGIF1. Previous studies have demonstrated that GRF proteins interact with GIF proteins to form functional transcriptional complexes. We speculate that CaGRF2 and CaG-IF1 may similarly assemble into a transcriptional complex (**Figure 6**). Combined with quantitative data, we hypothesize that the interaction between CaGRF2 and CaGIF1 could enhance genetic transformation efficiency in pepper.

## 3 Discussion

GRF transcription factors were plant-specific proteins regulating growth by maintaining cell division. Recent studies have increasingly characterized their functions and mechanisms in organ development (Baulies et al., 2025; Wu et al., 2021). GIF proteins function as transcriptional coactivators that form functional complexes with GRFs to enhance their regulatory activity. Although GRF-GIF interactions have been well documented to improve genetic trans-formation efficiency in multiple plant species (Zhao et al., 2025; Swinnen et al., 2025), their roles in pepper remain unexplored.

In this study, a total of 31 *GRF* genes and 9 *GIF* genes were identified across three solanaceous plants (**Tables 1**, **2**; **Supplementary Tables S1**–**S4**). The number of *GRF* genes in pepper was significantly reduced compared to those in tomato and tobacco, which might be attributed to the loss of certain genes during evolution due to environmental factors or other selective pressures. Elucidating the diversity of gene structures could provide a foundation for investigating the relationships among structure, evolution, and function (Xu et al., 2012). Analysis of the physicochemical properties of *GRF* and *GIF* genes in the three plant species revealed that these genes were relatively conserved in terms of exon number, protein size, and isoelectric point.

Phylogenetic analysis revealed that the *GRF* genes were classified into seven clades in tomato and tobacco, but only five clades in pepper (**Figure 1A**). This contrasts with the six-clade organization consistently reported in *Arabidopsis thaliana*, ginseng, Astragalus and wheat (Wang et al., 2023a; Zan et al., 2020; Wang et al., 2024). We speculated that this divergence may result from gene family expansion or contraction during the evolution of these solanaceous species, potentially as an adaptive response to specific environmental pressures. Such genomic modifications could enhance environmental adaptability while meeting the requirements for plant growth, development, and metabolic regulation. The *GIF* gene family was divided into two subfamilies, consistent with previous reports in other species (**Figure 1B**) (Chen et al., 2024).

The expansion of plant gene families occurs through whole-genome duplication (WGD) and tandem duplication events, which serve as key drivers of plant evolution and facilitate the diversification of gene functions (Doyle et al., 2008). In the *GRF* gene family, we identified two duplication events in pepper, five in tomato, and one in tobacco (**Figures 3A–C**). For the *GIF* gene family, only one duplication event was identified in tomato (**Figure 3E**). Furthermore, synteny analysis between pepper and tomato revealed 11 orthologous gene pairs in the *GRF* gene family and 3 in the *GIF* gene family (**Figures 3D** and F). The Ka/Ks ratio, which serves as an indicator of selective pressure on genes (Sun et al., 2017), revealed that both *GRF* and *GIF* gene families have undergone purifying selection (**Table 3**). These findings collectively suggest that these genes may perform conserved functional roles in plant development and adaptation (Hurst, 2002). These findings provide a crucial theoretical foundation for future investigations into the functional and evolutionary dynamics of *GRF* and *GIF* gene families.

The promoter, located upstream of a gene, is a DNA sequence containing numerous cis-acting elements. Its primary function is to regulate gene expression, while also serving as binding sites for various transcription factors and regulatory proteins (Hernandez-Garcia and Finer, 2014). Promoter cis-acting element analysis of GRF and GIF gene families in three Solanaceous plants identified abundant hormone and stress responsive motifs (**Figure 4**), indicating their functional roles in hormone signaling, plant growth regulation, and stress response, which is consistent with previous reports (Zhang et al., 2020; Xu et al., 2025).

Previous studies have demonstrated that *GRF* and *GIF* genes can enhance plant transformation efficiency (Debernardi et al., 2020; Li et al., 2024; Vandeputte et al., 2024). The genetic transformation system in pepper remains technically challenging. To investigate potential genetic regulators of pepper regeneration, we analyzed expression patterns of *GRF* and *GIF* gene families during the regeneration process. Notably, all *GRF* family members except *GRF5* showed significant upregulation. Among *GIF* genes, both *GIF1* and *GIF3* were markedly upregulated, while *GIF2* was undetectable (**Figure 5**). These findings strongly suggest that *GRF* and *GIF* gene families played crucial roles in pepper genetic transformation, providing a molecular foundation for optimizing pepper transformation protocols.

The regulatory relationship between *GRF* and *GIF* genes was complex, as they could physically interact to form functional transcriptional complexes that modulate gene ex-pression (Kim and Tsukaya, 2015). In the predicted protein-protein interaction network, CaGRF2 was found to interact with CaGIF1, though their precise functional mechanisms require further investigation (**Figure 6**). Elucidating how GRF and GIF proteins coordinately regulate gene expression may facilitate the development of novel strategies to enhance pepper growth and genetic transformation efficiency.

Although this study, through bioinformatic analysis and expression profiling, has preliminarily revealed the potential roles of *GRF* and *GIF* genes in the genetic transformation of pepper, further functional validation experiments are still required to elucidate their precise regulatory mechanisms. We plan to utilize the GRF-GIF complex (an interacting combination validated by yeast two-hybrid assays) to establish a stable genetic transformation system for overexpression or gene editing in peppers, directly verifying the impact of these genes on regeneration efficiency. This will provide strong evidence for elucidating the functions of GRF and GIF genes in the genetic transformation of peppers.

## 4 Conclusions

Genome-wide analysis identified 31 *GRF* and 9 *GIF* genes across three Solanaceae species. Phylogenetic reconstruction revealed 7 distinct clades for *GRFs* and 2 conserved clades for *GIFs*, with evolutionary evidence showing these gene families underwent significant expansion through duplication events followed by purifying selection. Promoter analysis identified abundant hormone- and stress-responsive cis-elements in both gene families. Expression profiling during pepper regeneration showed significant upregulation of all members except *GRF5* and *GIF2*. Protein interaction prediction further suggested that CaGRF2-CaGIF1 may form a functional transcriptional complex potentially crucial for genetic transformation processes.

## 5 Materials and methods

### 5.1 Identification of the GRF and GIF proteins in pepper, tomato, and tobacco

Genomic databases for tomato and tobacco were downloaded from the Gramene database (https://www.gramene.org/, accessed on 20 January 2025). Genomic databases for chili peppers were downloaded from the Gramene database (https://solgenomics.net/, accessed on 20 January 2025). Genomic databases for potato were downloaded from the Sol Genomics Network (https://solgenomics.net/, accessed on 7 November 2025). AtGRF1 and ZmGRF1 were used to identify similar protein sequences in pepper, tomato, and tobacco. The presence of the GRF-associated structural domains QLQ (PF08880.14) and WRC (PF08879.13) in all GRF protein sequences was verified using the Pfam website (http://www.ebi.ac.uk/Toolss/pfa/pfamscan/, accessed on 20 January 2025). AtGIF1 and ZmGIF1 were used to identify similar protein sequences of pepper, tomato and tobacco, and the same method was used to verify the presence of the GIF-related structural domain SSXT (PF05030.15) in all GIF protein sequences.

### 5.2 Protein sequence analysis, chromosomal locations, and gene structure

Using the ExPasy ProtParam tool (https://web.expasy.org/protparam/, accessed February 10, 2025) (Gasteiger et al., 2003). Predict the isoelectric point (pI) and molecular weight of the re-sulting sequence. Prediction of subcellular localization of the resulting proteins using the WoLF-PSORT tool (https://wolfpsort.hgc.jp/, accessed on 10 February 2025)) (Horton et al., 2007). Determining the exon/intron structure of GRF and GIF genes using the Gene Structure Display Server (GSDS2.0: http://gsds.cbi.pku.edu.cn/, accessed on 12 February 2025) (Hu et al., 2015). The ensemble plant database (http://plants.ensembl.org/index.html, accessed on 12 February 2025) was used to find the chromosomal locations of the *GRF* and *GIF* genes.

### 5.3 Protein conserved structural domains and motif analysis

From NCBI (https://www.ncbi.nlm.nih.gov/Structure/bwrpsb/bwrpsb.cgi, accessed January 25, 2025) validation of protein structural domains of GRF and GIF. Ten conserved motifs for GRF, five conserved motifs for GIF were queried via MEME (https://meme-suite.org/meme/, accessed on 27 January 2025).

### 5.4 The cis−acting elements in the promoter of *GRF* and *GIF* genes

The cis-elements in the promoter 2000 bp upstream of ATG in *GRFs* and *GIFs* were analyzed by PlantCARE (http://bioinformatics.psb.ugent.be/webtools/plantcare/html/) (Lescot et al., 2002), finding homeopathic action elements related to hormones and resistance and the remaining elements were mapped by TBtools.

### 5.5 Duplication analysis

Genomic analysis was performed using TBtools (v2.210), where the OneStep MCscanX-SuperFast function was employed to examine genome databases and gene annotation files from the three target species. Subsequently, Ka/Ks analysis was conducted on identified *GRF-GIF* gene pairs to determine their evolutionary relationships (Chen et al., 2023). The File Merge for MCscanX function in TBtools (v1.098726) was utilized to analyze syntenic relationships among *GRF* and *GIF* genes across the three species, facilitating evolutionary comparisons.

### 5.6 Protein interaction network prediction

Protein-protein interaction networks were predicted using the STRING database (https://string-db.org/, accessed on 20 February 2023) (Szklarczyk et al., 2015)and visualized using Cytoscape software (v3.9.1).

### 5.7 Plant materials, growth conditions, and treatments

Zunla-1 was selected for the experiment. Pepper seeds were soaked for 2h, washed with 2.5% concentration of NaClO for 20 min, and washed with sterile water for 5–6 times for 3min each time. At the end of the disinfection, the seeds were blotted dry with filter paper and placed in Sowing medium (4.4g/L MS, 30g/L sucrose, 8g/L Agar) for germination (28°C, dark). The cotyledons were cut into 0.5–1 cm explants and placed with the adaxial side up on the shoot induction medium (4.4g/L MS, 30g/L sucrose, 5mg/L 6-BA, 2mg/L IAA;8g/L Agar). They were cultured under conditions of 28°C with a 16/8-hour light/dark photoperiod for two weeks before sampling. The medium used is shown in **Supplementary Table S8**.

### 5.8 qRT–PCR

Total RNA was isolated using the RNA Easy Fast Plant Tissue Kit (DP452; TIANGEN, Beijing, China), followed by cDNA synthesis with HiScript III Reverse Transcriptase (R302; Vazyme, Nanjing, China). *CaGAPDH* (Yuan et al., 2021) and *CaActin2* (Ma et al., 2021) were used as internal reference genes, and the primer sequences are available at **Supplementary Table S9**. The qRT-PCR is based on the reported (Fei et al., 2024). Each value represents the mean of three biological replicates.

### 5.9 Tissue-specific expression pattern analysis

Transcriptome data for pepper were retrieved from the Pepper Full-length Transcriptome Variation Database (PFTVD 1.0) (http://pepper-database.cn/) (Liu et al., 2024). The expression levels of each gene in the GRF and GIF gene families in root, stem, leaf, flower, fruit, and seed tissues were obtained. A gene expression heatmap was subsequently generated using TBtool.

## Data Availability

The original contributions presented in the study are included in the article/**Supplementary Material**. Further inquiries can be directed to the corresponding authors.
