# Supplementary material for: Genome-wide identification of growth−regulating factor and GRF−interacting factor gene families across three Solanaceae species with functional analysis in pepper regeneration
Source: Front Plant Sci. 2025 Dec 10;16:1684045. doi: 10.3389/fpls.2025.1684045 (PMC12728049; doi:10.3389/fpls.2025.1684045)
Supplement: Supplementary file 1 [file DataSheet1.pdf]

Supplementary Material

1 Supplementary Figures and Tables

1.1 Supplementary Tables

Supplementary Table 1. Information of GRFs in tomato.

Gene Name	GeneID	Chr.	Exon Number	CDS (bp)	Protein length (aa)	Theoretical pI	Mw(KDa)	Subcellular localization
SlGRF1	*Solyc04g077510.3.1*	4	4	1788	595	8.3	64	Nucleus
SlGRF2	*Solyc02g092070.3.1*	2	5	1707	568	8.52	61	Nucleus
SlGRF3	*Solyc09g009200.2.1*	9	4	921	306	9.53	33	Nucleus
SlGRF4	*Solyc10g083510.2.1*	10	5	1170	389	9.18	42	Nucleus
SlGRF5	*Solyc07g041640.3.1*	7	3	1029	342	9	39	Nucleus
SlGRF6	*Solyc12g096070.2.1*	12	4	1044	347	7.13	38	Nucleus
SlGRF7	*Solyc08g075950.2.1*	8	6	1086	361	8.43	39	Nucleus
SlGRF8	*Solyc08g005430.3.1*	8	5	891	296	9.07	32	Nucleus
SlGRF9	*Solyc08g068760.1.1*	8	1	468	155	8.84	17	Nucleus
SlGRF10	*Solyc03g082430.1.1*	3	4	1377	458	5.97	50	Nucleus
SlGRF11	*Solyc08g083230.2.1*	8	3	1305	434	8.09	47	Nucleus
SlGRF12	*Solyc08g079800.3.1*	8	4	501	166	8.86	19	Nucleus

Supplementary Table 2. Information of GRFs in tobacco.

Gene Name	GeneID	Chr.	Exon Number	CDS (bp)	Protein length (aa)	Theoretical pI	Mw(KDa)	Subcellular localization
NtGRF1	*OIT21598*	scaffold04234	4	1818	605	8.41	65	Nucleus
NtGRF2	*OIT37486*	scaffold00305	4	1833	610	8.1	65	Nucleus
NtGRF3	*OIT04193*	4	4	1188	395	8.21	43	Nucleus
NtGRF4	*OIT03129*	5	4	1116	371	8.99	40	Nucleus
NtGRF5	*OIT35647*	scaffold00531	4	981	326	8.72	37	Nucleus

NtGRF6	*OIT32768*	scaffold00999	4	1110	369	8.64	42	Nucleus
NtGRF7	*OIT39313*	scaffold00119	4	1050	349	7.58	39	Nucleus
NtGRF8	*OIT32536*	scaffold00999	3	1071	356	7.67	39	Nucleus
NtGRF9	*OIS97502*	10	4	975	324	8.87	36	Nucleus
NtGRF10	*OIT08245*	1	4	1689	562	5.57	61	Nucleus
NtGRF11	*OIT29598*	scaffold01747	5	1428	475	6.53	51	Nucleus
NtGRF12	*OIT07825*	1	4	1239	412	9.17	45	Nucleus

Supplementary Table 3. Information of GIFs in tomato.

Gene Name	GeneID	Chr.	Exon Number	CDS (bp)	Protein length (aa)	Theoretical pI	Mw (KDa)	Subcellular localization
SlGIF1	*Solyc11g006230.2.1*	11	4	669	222	5.93	23	Nucleus
SlGIF2	*Solyc04g009820.3.1*	4	4	627	208	6.1	22	Nucleus
SlGIF3	*Solyc10g009280.3.1*	10	4	603	200	6.02	21	Nucleus
SlGIF4	*Solyc03g082480.3.1*	3	5	429	142	5.26	15	Nucleus

Supplementary Table 4. Information of GIFs in tobacco.

Gene Name	GeneID	Chr.	Exon Number	CDS (bp)	Protein length (aa)	Theoretical pI	Mw (KDa)	Subcellular localization
NtGIF1	*OIT19431*	scaffold07379	4	624	207	6.2	22	Nucleus
NtGIF2	*OIT04699*	3	4	624	207	5.86	22	Nucleus

Supplementary Table 5. Motif sequences of the GRF gene family

Motif	Sequences
Motif1	DPEPGRCRRTDGKKWRCSRDAVPDQKYCERHMHRGRNRSRK
Motif2	FTASQWQELEHQALIYKYLVAGMPVPPDLLLPIRKSLS
Motif3	SFQNMPLYSVANSEGLNYGSTATKLQMEPVSYGIDNKDYRY
Motif4	KSEQHPMRHFFDEWPTAQESW
Motif5	LDEGSNKNNFSTTQLSISIPMAPSDFSSR
Motif6	ISARFFHHPSLGYCSYYGKKF

Motif7	QMGLGIGTIIGEHNQKTGNWIPISWENSLGGPLGEVLHSTA
Motif8	WVLPSQVSSSPMARPKNDSQLLGSSTQMHLPNLLEPMIDSTISKR
Motif9	PVESQSTSQSLSTSMSHITTGSSNTSGSF
Motif10	ALNLATEGWDRSPRMGSSPTGVLQKSSFGSLSNSSAGSSPR

Supplementary Table 6. Motif sequences of the GIF gene family

Motif	Sequences
Motif1	TTDHIQQFLDENKSLILKIVESQNSGKLSECAESQAKLQRNLMYLAAIAD
Motif2	MQMQPMMAAYYPNNV
Motif3	MQMQPMMAAYYPNNV
Motif4	SQPQPPSMHSQLASGGMMQPGAHYLQQQQ
Motif5	GGFHDFGR

Supplementary Table 7. The sequence of cis-acting element analysis

Cis-acting element	Sequence
ABRE: abscisic acid responsiveness	ACGTG
ABRE: abscisic acid responsiveness	AACCCGG
GARE-motif: gibberellin-responsive element	TCTGTTG
P-box: gibberellin-responsive element	CCTTTTG
TATC-box: gibberellin-responsiveness	TATCCCA
TGA-element: auxin-responsive element	AACGAC
CGTCA-motif: MeJA-responsiveness	CGTCA
TGACG-motif: MeJA-responsiveness	TGACG
TCA-element: salicylic acid responsiveness	CCATCTTTTT
AuxRR-core: auxin responsiveness	GGTCCAT
TC-rich repeats: defense and stress responsiveness	ATTCTCTAAC
GT1-motif: light responsive element	GGTTAA
Sp1: light responsive element	GGGCGG

MRE: light responsiveness	AACCTAA
ACE: light responsiveness	CTAACGTATT
MBS: drought-inducibility	CAACTG
LTR: low-temperature responsiveness	CCGAAA

Supplementary Table 8. Culture medium formulation.

Culture medium	MS (g/L)	Sucrose (g/L)	6-BA (mg/L)	IAA(mg/L)	Agar(g/L)
Sowing	4.4	30	/	/	8
Shoot regeneration	4.4	30	5	2	8

Supplementary Table 9. Primers used in this study

Gene ID	Sequences
CaGIF1-qF	AGCTTCTGGTGGGATGATGC
CaGIF1-qF	TGCTGCTGCTGTTGTCCATA
CaGIF2-qF	GCACCAACAAGCCCAACAAA
CaGIF2-qF	GAAAACCAGTGCTTCCACCG
CaGIF3-qF	TAGTCCACCACCATCACCGA
CaGIF3-qF	TGCTCCGTTGTGACATTGTG
CaGRF1-qF	AAATGGCGGTGCTCAAGAGA
CaGRF1-qF	ATGTTCATCAGCTTGGCGGA
CaGRF2-qF	TCTCGTGGCAAATGTTCCGA
CaGRF2-qF	TTTCCATCTGTTCGACGGCA
CaGRF3-qF	GTTTCCAAACTAGCGGCAGC
CaGRF3-qF	GACAGGCTCCTTCTGCAACT
CaGRF4-qF	GAGTTACGGAAGCACCACGA
CaGRF4-qF	TCGTCATCAGCATCAGGAGC
CaGRF5-qF	CTTCTGGAGCTTCACACGGT
CaGRF5-qF	AGGTGCCACATCTCTTGAGC
CaGRF6-qF	CCATGCCTGTTCCTCCTCAG
CaGRF6-qF	AGCACCTCCATTTCTTCCCG
CaGRF7-qF	CTTGGGTGACGGTGGAATCT
CaGRF7-qF	CACATCCCTTCTGCACCTCC
GAPDH-qF	GGCTGCCATTAAGGAGGAGTCTG

GAPDH-qR	ACCACTCGCGTGCTGTAACCC
CaUBI3-qF	TGTCCATCTGCTCTCTGTTG
CaUBI3-qR	CACCCCAAGCACAATAAGAC

1.2 Supplementary Figures

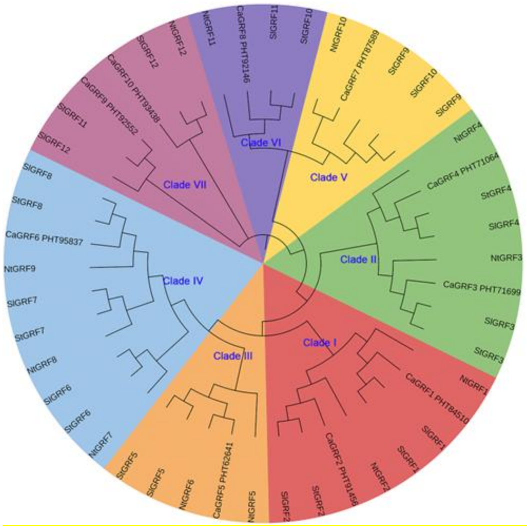

Supplementary Figure 1. Phylogenetic analysis of GRF proteins. Capsicum annuum genome (ASM51225v2, from Gramene-Ensembl).

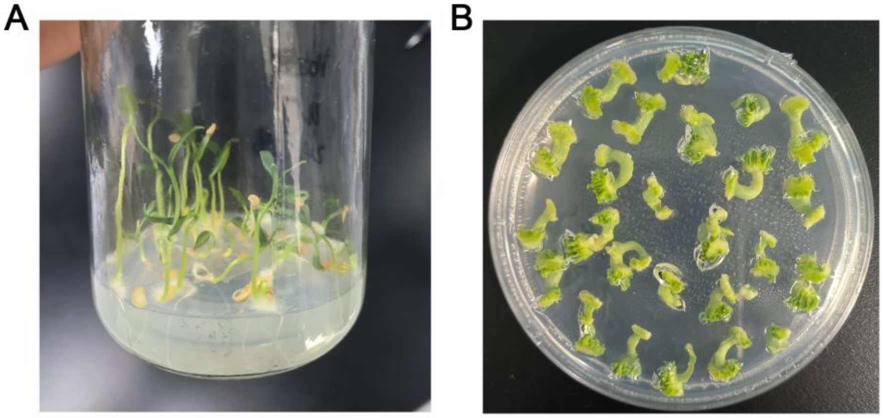

Supplementary Figure 2. Cotyledons and callus tissues in pepper. A. Cotyledons from approximately 10-day-old seedlings. B. Callus tissues after 2 weeks on shoot regeneration medium.

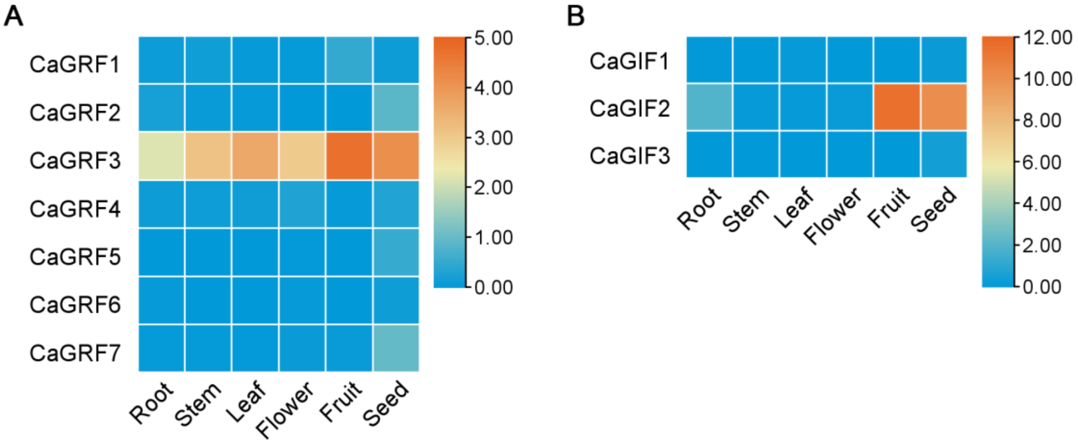

Supplementary Figure 3. Analysis of tissue expression patterns of *CaGRFs* and *CaGIFs.* A. Tissue expression pattern of *CaGRFs*. B. Tissue expression pattern of *CaGIFs*.
